# Editorial: Bone Marrow Adipose Tissue: Formation, Function, and Impact on Health and Disease

**DOI:** 10.3389/fendo.2017.00112

**Published:** 2017-05-29

**Authors:** William P. Cawthorn, Erica L. Scheller

**Affiliations:** ^1^University/British Heart Foundation Centre for Cardiovascular Science, The Queen’s Medical Research Institute, University of Edinburgh, Edinburgh, United Kingdom; ^2^Division of Bone and Mineral Diseases, Department of Medicine, Washington University, Saint Louis, MO, United States

**Keywords:** bone marrow adipocytes, bone marrow adipose tissue, bone remodeling, osteoporosis, bone marrow stromal cell, endocrinology and metabolism, bone metastases, multiple myeloma

**The Editorial on the Research Topic**

**Bone Marrow Adipose Tissue: Formation, Function, and Impact on Health and Disease**

Enthusiasm surrounding bone marrow (BM) adiposity has accelerated in recent years, motivated by numerous factors: adipocytes are abundant within the BM, BM adiposity increases in diverse pathophysiological states, and BM adipocytes can exert diverse effects both within and beyond the skeleton. This diversity has attracted a broad cross-section of scientists to the field and we are delighted to showcase their work in this special edition of *Frontiers in Endocrinology*.

## What’s in a Name?

Bone marrow adipocytes (BMAs) are large, roughly spherical cells containing a unilocular lipid droplet. They have gone by many names. Early work aptly recognized that BM has different colors, red or yellow, depending on its location—thus the terms *red marrow* and *yellow marrow* came into use (1). Red marrow consists of blood-forming cells with scattered adipocytes, whereas yellow marrow is filled almost entirely with adipocytes. It was not until 1950s–1960s that BM adipocytes became recognized as a fat depot with the potential for adipose tissue-like characteristics (2, 3). This caused the term *yellow marrow* to be replaced by “*marrow fat”* or “*fatty marrow”*. Even today, radiological studies still refer to the “bone marrow fat fraction” (BMFF) as a measure of marrow lipid content (Cordes et al.); however, as marrow adipocytes gain more recognition as a distinct, functional cell type, such designations are expanding beyond references to simple lipid. Herein, several options and abbreviations are introduced, including “BMAs” (Hardouin et al.; Ghali et al.,), “marrow adipose tissue” (MAT) (Scheller et al.; Walji et al.; Pino et al.; Sulston et al.), “bone marrow adipose tissue” (BMAT) (Hardouin et al.), and the “constitutive” and “regulated” subtypes of these (i.e., cMAT/rMAT or cBMA/rBMA). Clearly, reaching a consensus on this nomenclature will be important in providing a consistent framework on which this burgeoning field can progress. Based on the publications to date and the overlap of the acronym “MAT” with “muscle adipose tissue”, we propose adopting “BMAT” to refer to the tissue and “BMA” to refer to the adipocytes therein.

## Origins and Expansion of BMAT

Bone marrow adipose tissue develops postnatally and accounts for 50–70% of BM volume in healthy adult humans; hence, BMAT development is a normal physiological process. BMAT further accumulates with aging and in diverse clinical conditions, suggesting pathological implications of aberrant BMAT formation and function. In their comprehensive review, Hardouin et al. further discuss these issues, which underscore the need to better understand BMAT in physiology and disease. Several other articles herein provide additional focus on BMAT developmental origins and the mechanisms underlying BMAT expansion. Tencerova and Kassem review the differentiation of BMAs from BM stromal cells (BMSCs), discussing lineage tracing studies that are identifying BMSCs committed to the adipogenic lineage; signaling pathways that regulate this commitment; and the heterogeneity of committed BMSC subpopulations. The latter is underscored by Jacobs et al., whose study reveals site-specific differences in BMSC adipogenic potential, while Lindenmaier et al. provide further evidence for the important role of leptin in regulating adipogenic and osteogenic differentiation of BMSCs. Elsewhere, Hamrick et al. discuss BMAT expansion by raising a compelling question: are there common mechanisms underlying adipocyte accumulation in muscle and bone? This unique perspective highlights the many conditions with similar effects on BMAT expansion and intramuscular adipocyte accumulation, and how these common mechanisms might be targeted for therapeutic benefit.

Several other papers in this Research Topic address BMAT expansion in adverse metabolic states. As reviewed by Ghali et al., increased BMAT during caloric restriction is now well established, although the causes and consequences of this remain incompletely understood. At the other end of the metabolic spectrum, studies by Scheller et al. and by Lindenmaier et al. provide additional evidence that, in mice, BMAT accumulates during obesity, and further reveal that this is prevented by leptin treatment or weight loss. This is reminiscent of exercise’s ability to prevent BMAT expansion during obesity or other conditions, as discussed by Pagnotti and Styner. Finally, Walji et al. reveal that, in *Mfap2^−/−^* mice, BMAT expansion coincides with the development of insulin resistance, but not of obesity or hyperglycemia.

Despite such progress, more research is needed to identify committed BMA progenitor(s) in humans and to clarify the mechanisms underlying gain or loss of BMAT in normal development and disease.

## BMAT—Good, Bad, or Somewhere in Between?

These questions regarding BMAT formation are related to a broader question: what is the function of BMAT? Given that it is a feature of normal anatomy and development, it would be surprising if BMAT did not fulfill at least some physiological functions. Yet, it also seems likely that, in adverse contexts, BMAT can contribute to disease pathogenesis. Notably, increased BMAT often coincides with decreased bone mass, suggesting that bone formation and marrow adiposity are linked. One possibility, touted throughout this Research Topic, is that a common BM progenitor undergoes adipogenesis at the expense of osteogenesis (Hardouin et al.; Pino et al.; Tencerova and Kassem; Jacobs et al.; Pagnotti and Styner). However, other contributors show that alterations in BMAT quantity do not always coincide with opposite changes in bone mass, whether in response to obesity (Scheller et al.), insulin resistance (Walji et al.), leptin treatment (Lindenmaier et al.), or caloric restriction/anorexia (Ghali et al.). Another possibility is that BMAs secrete factors to directly regulate bone remodeling, even without gross changes in BMAT quantity. Other potential secretory functions of BMAT are further explored by Sulston et al., who investigate BMAT as a source of the hormone adiponectin. Such endocrine properties might allow BMAT to act outside the skeleton, exerting systemic effects on metabolism and beyond.

A final concept gaining increasing attention is that BMAs modulate tumor growth and metastasis. Morris and Edwards provide a comprehensive review, while Falank et al. offer an in-depth focus on the interplay between BMAT and multiple myeloma cells. Complementing these articles, Herroon et al. describe novel methods for studying interactions between BMAs and prostate metastases. Together, these articles emphasize that some BMA-derived factors can stimulate tumor progression, while other secretory products can exert inhibitory effects. Targeting these mechanisms may thereby represent a novel therapeutic avenue in the battle against myeloid cancers and metastatic bone disease.

## Technological Advancements

Recent advances in quantitative imaging of BMAs have accelerated our understanding of this relatively inaccessible fat depot in both rodents and humans. In the article by Hardouin et al., the evolution of these techniques is described in detail, including magnetic resonance imaging, proton magnetic resonance spectroscopy, and high-resolution computed tomography. In humans, synthesis, adaptation, and modification of existing techniques have allowed researchers to non-invasively monitor BMAT development and expansion in parallel with changes to the healthy and diseased skeleton (reviewed in Cordes et al.). In rodents, imaging of skeletal BMAT with osmium tetroxide and computed tomography has emerged as the current gold standard, facilitating both volumetric quantification of BMAT and spatial analysis of BMAT patterning within bone. Longitudinal application of these techniques in rodents is leveraged in this edition to demonstrate that bone loss precedes BMAT expansion in two models of obesity and diabetes (Scheller et al.; Walji et al.), leading to the conclusion that, in this context, accumulation of BMAT may be more closely linked to peripheral adipose tissue dysfunction than bone turnover.

Unlike widespread advances in imaging, techniques for robust molecular analysis and genetic manipulation of BMAs are still lacking. However, the work by Herroon et al., presented in this edition, provides a glimpse of the future possibilities. The authors detail two novel *in vitro* approaches to study the interactions of primary BM-derived adipocytes and tumor cells in a three-dimensional coculture. Given the challenges inherent in studying BMAs within their native skeletal microenvironment, *ex vivo* and *in vitro* systems that recapitulate key aspects of BMAT biology will be crucial to elucidation of BMA function.

## Quality vs Quantity

Emerging evidence supports the concept that not all BMAs are created equal and that they have the potential for maladaptation with age and disease. Indeed, it may not be the total amount of BMAT, but rather its context-specific phenotype that dictates the relationships between BMAT, bone and whole-body metabolism. In this edition, our authors highlight the ability of BMAT to undergo pathological change in diseases such as osteoporosis (Ghali et al.). Morris and Edwards also discuss the relevance of this concept for tumor metastasis, which may explain context-specific tumor-promoting and tumor-suppressive effects of BMAT. A key aspect of this phenotypic switch or maladaptation appears to be shifts in the lipid composition of BMAs (discussed in Pino et al.). While the source of these differences remains unknown, current hypotheses suggest consideration of site-specific progenitor populations (Jacobs et al.) and microenvironmental programming of both developing and mature BMAs. Future work will undoubtedly be needed to explore the differences in BMAT between skeletal sites and to determine its implications for bone loss and metabolic health.

## Prospectus

This edition provides a comprehensive overview of the state of the field of BM adiposity, details novel hypotheses, and provides opportunities for development and growth. Based on the work presented, it is clear that future studies are warranted to define the biological functions of BM adipocytes—having implications for bone turnover, hematopoiesis, systemic metabolic homeostasis, tumor metastasis, energy storage, and beyond.

## Author Contributions

WC and ES jointly wrote this editorial. ES initially wrote the section entitled “What’s in a Name?”, with WC then contributing further edits. WC then focused on the sections entitled “Origins and Expansion of BMAT” and “BMAT—Good, Bad, or Somewhere in between?”, while ES focused on “Technological Advancements”, “Quality vs Quantity,” and the “Prospectus”.

## Conflict of Interest Statement

The authors declare that the research was conducted in the absence of any commercial or financial relationships that could be construed as a potential conflict of interest.
